# The association between online learning and young people’s participation in sports and its mediating mechanisms

**DOI:** 10.3389/fpubh.2025.1715379

**Published:** 2026-01-12

**Authors:** Fan Yang

**Affiliations:** Department of Physical Education, Sichuan International Studies University, Chongqing, China

**Keywords:** CFPS, mediating mechanisms, online learning, physical activity participation, youth

## Abstract

**Background:**

Against the backdrop of the concurrent Digital China and Healthy China strategies, pervasive digital technologies are profoundly reshaping how young people accumulate human capital and form health behaviors. As a salient form of human capital investment, online learning’s independent effect on sports participation remains unclear and may be obscured by the influence of formal education. Clarifying this relationship is important for understanding mechanisms of health behavior formation in the digital era.

**Methods:**

Using the China Family Panel Studies (CFPS) databases for 2020 and 2022, a pooled cross-sectional dataset comprising 8,530 young adults aged 18–44 was constructed. We estimated linear and logistic regression models in a stepwise fashion, systematically controlling for demographic characteristics, urban–rural residence, health status, education, and other factors to identify the net effect of online learning on sports participation. Heterogeneity was examined via subgroup regressions, and the mediating role of perceived “importance of learning” was tested using a bootstrap approach.

**Results:**

After rigorously controlling for educational attainment, online learning exhibits a significant positive effect on both the frequency of physical exercise and the probability of participation. Notably, years of education per se are consistently negatively correlated with engagement in physical exercise. The promotive effect of online learning is stronger among males and urban youth. Mediation analysis indicates that enhancing individuals’ perceived importance of learning significantly increases the indirect effect that promotes participation in physical activity.

**Conclusion:**

The findings indicate that online learning is an independent and effective form of digital human capital that promotes sports participation among youth. Its influence extends beyond information transmission to driving behavior change by shaping positive learning values. The study suggests that digital health promotion practice should leverage the cognitive activation function of online learning platforms and prioritize the development of high-quality digital health learning resources to sustain the cultivation of lifelong healthy habits among young people.

## 1 Introduction

Digital transformation has accelerated the deep integration of digital technologies with China’s economy and society, reshaping multiple dimensions of social operation. Under the synergistic influence of the national strategies “Digital China” and “Sports Power,” “online learning”—as an efficient, flexible, and individualized mode of acquiring knowledge and skills—represents a usage behavior oriented toward learning that may exert more positive and enduring effects on sports participation by enhancing individuals’ sports-related knowledge, imparting scientifically based exercise methods, and strengthening self-management capacities. Scholarly inquiry into the relationship between digital technologies and sports participation has produced valuable explorations and a body of theoretical and empirical findings from multiple perspectives. Research from the psychological perspective indicates that online media can influence exercise behavior by affecting individuals’ attitudes toward sports, motivation, and self-efficacy (1). From the communication studies perspective, scholars emphasize that online media expand the dissemination boundaries of sports information and diversify forms of sports-related social interaction, thereby facilitating the construction and maintenance of sports communities and mobilizing broader public participation in sports (2). Sociological research has focused on the positive role of online social interaction in accumulating sports-related social capital and promoting social integration (3).

However, extant studies exhibit divergent findings and notable limitations. Some research supports a “media mobilization” perspective, arguing that internet use exerts a significant positive effect on sports participation (4); other studies provide evidence for a “media displacement” view, contending that internet use may encroach on time for physical exercise or increase sedentary behavior, thereby producing negative effects (5, 6); still other investigations have failed to identify a significant association between the two (7). These perspectives have not adequately accounted for the interaction between macro-level social structural factors and micro-level individual characteristics, and thus struggle to capture the complexity and heterogeneity of internet effects. Accordingly, this study uses the CFPS database and treats “online learning” as the primary explanatory variable to examine its impact on youth sports participation, thereby furnishing more precise evidence for understanding the intrinsic mechanisms through which digitalization empowers youth sports development. Assessing the value of digital instruction in promoting youth sports development is of critical importance for designing targeted and effective strategies to increase youth participation in physical activity.

## 2 Literature review

“Online learning” refers to the process by which individuals utilize internet technologies and digital platforms to acquire knowledge, skills, or information, and achieve internalization of knowledge and enhancement of abilities through interaction, practice, and other activities. Its core characteristics are technological dependence, temporal and spatial flexibility, and resource accessibility (8). Operationally, international research often defines online learning along two dimensions: behavioral participation and goal orientation (9); domestic scholars, situating the concept within the context of educational informatization, emphasize the integrative nature of online learning scenarios, arguing that it not only encompasses narrow course-based study but also includes fragmented acquisition of sport-related knowledge via social media and short-video platforms (10). Notably, during the COVID-19 pandemic, online learning was selected as the preferred means to continue education in the pandemic era, and its overall performance exceeded expectations (11).

With respect to the effectiveness of online learning, an empirical analysis of large-scale online instruction that focused on the interaction between information literacy and engagement in online learning found a positive correlation between university students’ information literacy and their level of learning engagement, with different dimensions of information literacy exerting varying degrees of influence on the different components of online learning engagement (12). In analyses of determinants of continued participation in emerging sports, media interest was found to exert a significant effect on attitude, which, together with subjective norms and perceived behavioral control, influenced behavioral intention (13). Concurrently, the Ministry of Education of China has accelerated the development of online courses, and in recent years has established a large number of high-quality online courses, thereby promoting young people’s media interest in acquiring knowledge.

The World Health Organization defines youth as individuals aged 18–44 years. Engaging in regular physical activity during youth improves cardiorespiratory and muscular health, skeletal and cardiac metabolic health, and many of these benefits persist into adulthood. Furthermore, physical activity exerts positive effects on cognitive development and prosocial behavior (14). Existing research has largely focused on group differences in youth sports participation, revealing a significant “gender–education” interaction effect on participation (15). Systematic reviews further indicate that, within youth populations, team sports participation is more effective than individual sports at promoting sustained engagement; social media empowerment facilitates and reinforces adolescent sports-related health promotion; self-empowerment manifests as autonomy and self-regulation; family empowerment reflects guidance and trust; and school empowerment is evident in organizational actions and advocacy (16, 17).

Under the dual impetus of the “Healthy China” strategy and the Education Informatization 2.0 Action Plan, the role of online learning in promoting youth participation in physical activity has been corroborated across multiple dimensions. From a resource-access perspective, high-quality online sports resources can effectively bridge young people’s gaps in sports-related knowledge—research based on a national youth sports health survey found that youths who use the Internet to learn about sports injury prevention engage in physical activity 1.5 times more per week than nonusers (18). From a skills-improvement perspective, user management and knowledge management within online educational knowledge-sharing communities provide the methodological and procedural support for such capacity building (19). Regarding group heterogeneity, young women and youths with lower educational attainment represent the primary beneficiary groups of the promotional effects of online learning.

Based on social cognitive theory, online learning can indirectly promote physical activity among youth by enhancing their “exercise self-efficacy” and “health cognition.” Using survey data collected during the COVID-19 pandemic, found that information literacy (including information acquisition and application skills) indirectly improved young people’s attitudes toward physical activity by increasing engagement in online learning; the mediating effect attributable to “information application ability” accounted for 31.7%—specifically, after acquiring exercise skills through online learning, youths’ confidence in their own athletic abilities increased (self-efficacy improved), which in turn made them more willing to participate in physical activities (20). From the perspective of time-use theory, youths’ time resources are limited, and online learning and participation in physical activity may exhibit a trade-off relationship; however, this relationship can be moderated by habit formation: if youths develop a composite habit of “online learning + physical exercise,” the efficiency of time use can be significantly enhanced (15).

At the levels of cognition and bodily behavior, a biological perspective has been used to explore how bodily signals influence the brain’s regulation of intrinsic functions, positing that the brain–body interaction, which dissolves the classical separation between “lower-level” bodily functions and “higher-level” cognitive functions (such as learning and memory), promises substantial gains (21). Quantification of systematic and random measurement error underscores the importance of providing sufficiently habituation-based regimens in neurocognitive tasks (22). Adequate practice of physical activity affords individuals extraordinary possibilities for learning, thereby enhancing engagement in training and developmental processes (23). Compared with students who exhibit lower perceptual abilities, those with higher collaborative perceptual levels display more comprehensive and diversified cognitive engagement and attain superior learning outcomes (24). Moreover, cognition and behavior are mutually reinforcing: cultivating balanced sleep and exercise habits can strengthen perceptions of autonomy, competence, and relatedness among students with lower average GPAs in online physical education courses (25).

Population heterogeneity significantly moderates the effect of online learning on youth sports participation. Participation in physical education classes is positively associated with adolescents’ physical activity levels across gender and age groups (26). By gender, female youths are more inclined to obtain knowledge about low-intensity exercise through online learning, whereas male youths tend to consume online content oriented toward high-intensity exercise (9). By educational attainment, youths with a bachelor’s degree or higher demonstrate superior information-filtering capabilities and can more efficiently acquire high-quality sports resources via online learning; consequently, the positive effect of online learning on their sports participation is more pronounced (12). Urban–rural differences and the accessibility of sports facilities further exacerbate heterogeneity in the impact of online learning. Research by Lu and Xiao-lin indicates that urban youths, benefiting from more complete offline sports facilities, can translate exercise skills acquired online into actual participation more rapidly (27). However, analyses based on CFPS panel data show that, as rural internet infrastructure improves, the promoting effect of online learning on rural youths’ sports participation has been increasing year by year (28). When sports venues are available within a 1 km radius of youths’ residences, the facilitating effect of online learning on sports participation is significantly stronger than for those without nearby venues. Socioeconomic status positively predicts organized sports participation among children and adolescents (29).

Based on the foregoing analysis, this study therefore proposes the following research hypothesis:

*H1*: Online learning has a significant positive effect on adolescents’ participation in physical activity.

*H2*: Cognition enhances the promoting effect of networked learning on participation in physical activity.

*H3*: Education may amplify the effect of online learning on physical activity participation.

*H4*: The effect of online learning on physical activity participation exhibits significant group heterogeneity.

## 3 Materials and methods

### 3.1 Data sources

The data for this study are drawn from the public-release dataset of the China Family Panel Studies (CFPS). Implemented by the Institute of Social Science Survey (ISSS) at Peking University, CFPS aims to capture changes in Chinese society, economy, demography, education, and health by tracking data at individual, household, and community levels. CFPS provides robust empirical support for examining the impact of online learning on young people’s participation in physical activity. This study employs individual-level data from 2020 and 2022 and constructs a balanced panel of individuals aged 18–44 by merging the two waves. After merging, systematic data cleaning and variable reconstruction were conducted to ensure comparability of variables across years. The final analytic sample, which includes complete information on educational variables, comprises 8,530 observations—4,094 from 2020 and 4,436 from 2022. Data were analyzed using Stata 18.0.

### 3.2 Variable selection

#### 3.2.1 Dependent variable

The dependent variable in this study is youth sports participation. It is derived from the individual questionnaire item “QP701N Frequency of exercise (times),” measured as the number of exercise days per week and defined as “sports_freq,” using the raw reported value. Weekly total exercise time (weekly_exercise_time) was calculated by multiplying exercise frequency by the minutes reported in “QP702N Exercise duration (minutes)” for each session. Following the WHO guideline recommending 150–300 min of moderate-intensity physical activity per week for all adults (30), participants whose weekly_exercise_time reached at least 150 min and was not below the sample median were categorized as engaging in active sports participation (sports_active) and coded as 1; otherwise they were coded as 0, thereby distinguishing “active” from “non-active” sports participants.

#### 3.2.2 Core explanatory variable

The primary explanatory variable in this study is online learning participation (netlearn_basic), operationalized using the survey item “QU94: Do you engage in online learning?” with responses coded as 1 = yes and 0 = no. In addition, daily online learning (netlearn_daily) is defined based on qu941: “Do you engage in online learning every day?” and is coded similarly (1 = yes, 0 = no).

#### 3.2.3 Mediating variable

The process by which individuals engage in online learning is essentially one of receiving systematic information inputs and constructing a coherent knowledge system. Enhancement of intrinsic motivation arises from the satisfaction of basic psychological needs and serves as a core driving force in self-regulated learning (31, 32). Accordingly, we define a variable for perceived learning importance (learn_importance) based on the questionnaire item qu954, “How important do you consider learning to be for you?,” which captures the individual’s subjective evaluation of the importance of learning on a 1–5 scale. This variable is treated as a continuous variable with values ranging from 1 to 5. According to social cognitive theory’s mechanisms of observational learning and self-regulation, when individuals repeatedly experience and internalize the value of self-improvement through online learning, this perception of importance may transfer to the management of physical health and facilitate participation in physical activity.

#### 3.2.4 Control variables

**Figure 1:**
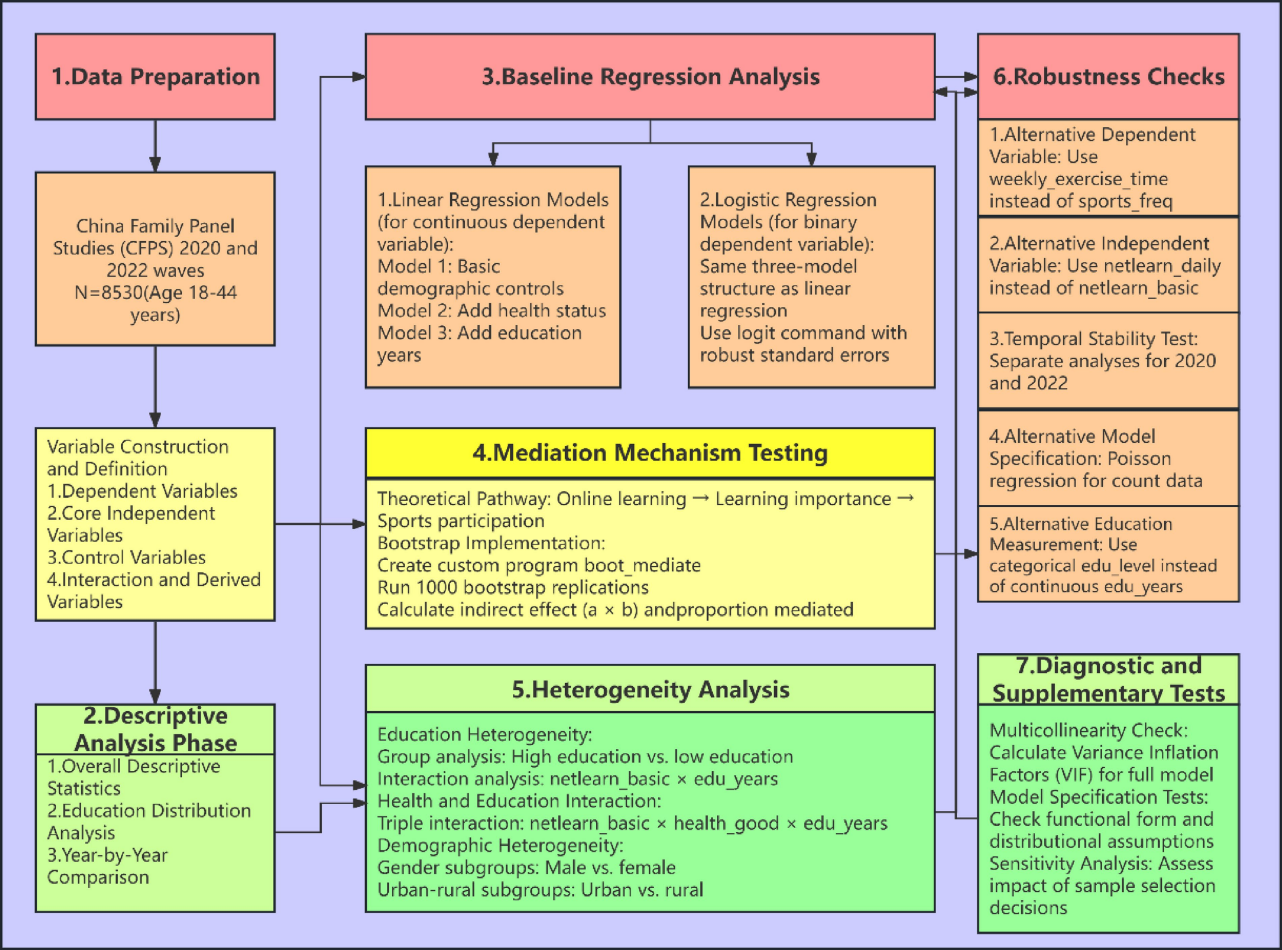
Research flowchart.

Education is one of the most fundamental determinants influencing nearly all socioeconomic behaviors of individuals. Basic differences in population composition, urban–rural residence, health status, and cohort (year) effects can all underlie behavioral heterogeneity; thus, including education as a control variable can effectively mitigate spurious associations between online learning and participation in physical activity. Human capital, broadly defined as the skills acquired through formal education (33), We incorporate years of schooling (edu_years) as a continuous variable in the baseline model to linearly account for this fundamental human capital disparity; edu_college (whether one has postsecondary education) and edu_level (7-category classification) are employed for subgroup regressions and categorical tests. In addition, age, sex, urban–rural status, and health status are included as control variables, together forming a comprehensive hierarchy that spans individual microcharacteristics, meso-level urban–rural context, and macro educational structure, and ultimately controls for time trends (see Figure 1).

### 3.3 Model construction

To comprehensively and rigorously assess the effect of online learning on sports participation, baseline regression models were first specified according to the different measurement types of the dependent variable, comprising linear regression and logistic regression models. The linear regression models were specified following a parsimonious-to-complex logic, with confounding variables introduced progressively across nested specifications. Both model types employed a consistent, stepwise control-variable strategy to systematically identify and account for potential confounders, with the ultimate aim of estimating the conditional causal effect of online learning on sports participation.

#### 3.3.1 Design of the regression model

A linear regression model is appropriate for the continuous dependent variable “weekly exercise frequency (sports_freq),” with its estimated coefficients directly interpretable as marginal effects. This model includes only the core independent variables, urban–rural variable, basic demographic covariates, and year fixed effects, and is intended to estimate the crude association between online learning and exercise frequency, serving as a baseline for subsequent model comparisons.


$$\begin{array}{c} \text{sports}\_{\text{freq}}_{i}=\beta_{0}+\beta_{1}{\text{NetLearn}}_{i}+\beta_{2}{\text{Male}}_{i}+\beta_{3}{\mathrm{Age}}_{i}\\ +\beta_{4}{\text{Urban}}_{i}+\delta \cdot {\text{Year}}_{i}+\varepsilon_{i} \end{array}$$

Next, include the health status variable (Health Good). Good health may underlie both higher academic engagement and greater participation in intensive physical activity. If not controlled for, a “health selection” bias may arise. This step aims to isolate the potential influence of health capital on the estimation results.


$$\begin{array}{c} \text{sports}\_{\text{freq}}_{i}=\beta_{0}+\beta_{1}{\text{NetLearn}}_{i}+\beta_{2}{\text{Male}}_{i}+\beta_{3}{\mathrm{Age}}_{i}\\ +\beta_{4}{\text{Urban}}_{i}+\beta_{5}{\text{HealthGood}}_{i}+\delta \cdot {\text{Year}}_{i}+\varepsilon_{i} \end{array}$$

Finally, years of education (EduYears) were added as a control variable. Education, as one of the most important forms of human capital and a key antecedent, can profoundly influence individuals’ information access, health beliefs, and behavioral patterns. By introducing education last to form a fully controlled model, the primary objective is to test whether the independent effect of online learning remains robust after removing this powerful confounding pathway of formal education. This model is intended to provide an estimate that is as close as possible to the conditional causal effect.


$$\begin{array}{c} \text{sports}\_{\text{freq}}_{i}=\beta_{0}+\beta_{1}{\text{NetLearn}}_{i}+\beta_{2}{\text{Male}}_{i}+\beta_{3}{\mathrm{Age}}_{i}\\ +\beta_{4}{\text{Urban}}_{i}+\beta_{5}{\text{HealthGood}}_{i}+\beta_{6}{\text{EduYears}}_{i}\\ +\delta \cdot {\text{Year}}_{i}+\varepsilon_{i} \end{array}$$

A logistic regression model was employed for the binary dependent variable “sports_active”; its coefficients represent effects on the log odds of the event. The core analytical logic (stepwise control of confounders) mirrors that of the linear models. First, a baseline logistic regression model was estimated, corresponding to the L1 model in the linear sequence, intended to assess the association between online learning and the odds of meeting the “actively participating in sports” criterion while controlling only for basic demographic characteristics and year effects, thereby establishing an initial link. Second, health- and education-related covariates and other control variables were incrementally introduced to estimate the final logistic model, corresponding to the fully specified model in the linear sequence. The objective is to directly test, on the behavioral dimension of “whether one actively participates,” whether the net effect of online learning persists after comprehensive control for confounders, thus providing multidimensional corroboration of the linear regression findings.


$$\begin{array}{c} \log(\frac{P(\text{sports}\_{\text{active}}_{i}=1)}{1-P(\text{sports}\_{\text{active}}_{i}=1)})=\alpha_{0}+\alpha_{1}{\text{NetLearn}}_{i}+\alpha_{2}{\text{Male}}_{i}\\ +\alpha_{3}{\mathrm{Age}}_{i}+\alpha_{4}{\text{Urban}}_{i}\\ +\alpha_{5}{\text{HealthGood}}_{i}\\ +\alpha_{6}{\text{EduYears}}_{i}+\lambda \cdot {\text{Year}}_{i} \end{array}$$

#### 3.3.2 Mechanism testing models

To test the theoretical mechanism of “cognitive enhancement,” a structural equation model combined with the bootstrap method was employed to directly examine the theoretical causal chain “online learning → increased perception of the importance of learning → promoted physical activity.” Here, a_1 measures the effect of online learning on cognition, and b measures the effect of cognition on behavior. A significant indirect effect (a_1 × b) would provide empirical support for the hypothesis that online learning influences behavior by altering intrinsic motivation and health-related beliefs.


$$M_{i}(\text{LearnImportance})=a_{0}+a_{1}\text{NetLear}n_{i}+X_{i}\kappa +e_{i}$$

$$Y_{i}=c\prime \text{NetLear}n_{i}+bM_{i}+X_{i}\omega +v_{i}$$

## 4 Research findings

### 4.1 Descriptive statistics

Table 1 presents descriptive statistics for the analytical sample (*N* = 8,530). Half of young adults (50.7%) reported regular sports participation, with a mean frequency of 3.51 sessions per week (SD = 1.54), The mean weekly exercise duration was as high as 201.38 min, but the large standard deviation indicates substantial interindividual variability in exercise time commitment. For online learning, 44.9% had engaged in this activity. The average years of schooling was 12.30 (SD = 3.45); 40.58% had tertiary education or above, while 59.42% completed high school or below. Only 7.5% self-rated their health as good. Demographically, 55.1% were male, mean age was 29.96 years, and 37.9% held urban household registration. From 2020 to 2022, sports participation declined (53.3 to 48.2%), whereas online learning increased (42.0% to 47.6%), suggesting shifting behaviors in the post-pandemic period that merit further analysis.

**Table 1:** Descriptive statistics of key variables.

Variable	*N*	Mean	SD	Min	Max	Description/measurement
Dependent variables						
Sports_active (1 = yes)	8,530	0.507	0.500	0	1	Binary: active sports participation
Sports_freq	8,530	3.510	1.541	1	7	Times per week (ordinal, 1–7)
Weekly exercise time	8,530	201.384	180.112	1	2,100	Minutes per week
Core independent variable						
Online learning (1 = yes)	8,530	0.449	0.497	0	1	Binary: participated in online learning
Education variables						
Years of schooling	8,530	12.299	3.450	0	22	Continuous (years)
Tertiary education (1 = yes)	8,530	0.406	0.491	0	1	Binary: junior college or above
Control variables						
Male (1 = yes)	8,530	0.551	0.497	0	1	Binary
Age	8,530	29.961	7.399	18	44	Years
Urban (1 = yes)	8,530	0.379	0.485	0	1	Binary
Good health (1 = yes)	8,530	0.075	0.264	0	1	Binary: self-reported health status is good
Year 2022 (1 = yes)	8,530	0.520	0.500	0	1	Binary (base: year 2020)

### 4.2 Regression analysis

#### 4.2.1 The significant promotive effect of online learning

Across all models, online learning exerts a significant positive effect on both exercise frequency and participation in physical activity. After controlling for all covariates in the linear regression, individuals who engaged in online learning exercised on average 0.145 times more per week than non-participants (*β* = 0.145, *p* < 0.01). In the logistic regression, estimated by average marginal effects, online learning increased the probability of participating in physical exercise by an average of 3.8 percentage points (AME = 0.038, *p* < 0.01). This effect remains highly significant across Models 1–3, and the coefficient substantially increases after the introduction of educational variables (the coefficient in the linear model rising from 0.072 to 0.145). These statistical findings corroborate the central hypothesis of this study: online learning can effectively promote young people’s participation in physical exercise. Online education platforms reduce technical barriers to exercise by providing accessible fitness knowledge and a variety of online courses.

#### 4.2.2 The moderating effect of years of education and urban–rural disparities

One salient finding is the consistent and robust suppressive effect of “years of education” on physical exercise. In the full model, each additional year of education is associated with an average decrease of 0.054 exercise sessions (*β* = −0.054, *p* < 0.01), and the probability of participation is also significantly reduced (coefficient = −0.025, *p* < 0.01). This provides empirical support for the “time squeeze” hypothesis: longer schooling typically entails more intensive cognitive labor and occupational pressures, which structurally encroach on individuals’ leisure time and motivation to exercise. The inclusion of the education variable also fundamentally alters the estimates for the “urban–rural household registration” variable. In Model 2, the “urban” indicator is not significant in the linear regression. However, after controlling for education (Model 3), urban hukou holders exhibit significantly higher exercise frequency (*β* = 0.089, *p* < 0.05) and participation probability (coefficient = 0.252, *p* < 0.01). This reversal indicates that educational disparities between urban and rural areas act as a strong confounder. Once the educational advantage is accounted for, the environmental advantages of urban areas—such as superior sports facilities and a stronger fitness culture—become evident.

#### 4.2.3 Mechanistic explanation for differences in variable significance

The differences in variable significance between linear and logistic models do not indicate model deficiencies, but rather stem from the two-stage decision nature characteristic of physical exercise behavior, offering a finer-grained perspective for understanding how sociodemographic factors exert influence. The variable “male” is highly significant in the logistic regression (*p* < 0.1), but not significant in the linear regression (*p* > 0.1), Indicates that gender differences primarily affect the decision threshold of “whether to participate” in exercise. Among those who do exercise, there is no significant difference between men and women in exercise frequency. Age exhibits a small positive effect in the linear regression (*p* < 0.05) but a consistently negative effect in the logistic regression (*p* < 0.01). This ostensibly contradictory pattern may reflect changes in exercise participation across the life course: overall participation rates decline with age—particularly during the transition from youth to middle age, potentially due to increased family and work responsibilities—whereas among individuals who continue to exercise, advancing age may be associated with more regular and stable routines, producing a modest increase in exercise frequency (see Table 2).

**Table 2:** Baseline regression results: comparison of linear and logit models.

Variable	Linear model (DV: exercise frequency)			Logistic model (DV: sports active)		
	Model 1	Model 2	Model 3	Model 1	Model 2	Model 3
Core predictor						
Online learning	0.072** (0.034)	0.072** (0.034)	0.145* (0.034)**	0.121*** (0.044)	0.121*** (0.044)	0.154* (0.045)**
Key controls						
Years of schooling			−0.054* (0.005)**			−0.025* (0.007)**
Good health		−0.042 (0.068)	−0.066 (0.068)		−0.059 (0.085)	−0.069 (0.084)
Demographics						
Male	0.035 (0.034)	0.035 (0.034)	0.036 (0.033)	0.362* (0.044)**	0.362* (0.044)**	0.363* (0.044)**
Age	0.002 (0.002)	0.002 (0.002)	0.005 (0.002)	−0.016* (0.003)**	−0.015* (0.003)**	−0.014* (0.003)**
Urban	−0.021 (0.034)	−0.021 (0.034)	0.089 (0.036)	0.202* (0.046)**	0.202* (0.046)**	0.252* (0.048)**
Year 2022 (ref: 2020)	−0.044 (0.033)	−0.044 (0.033)	−0.102* (0.034)**	−0.200* (0.044)**	−0.201* (0.044)**	−0.228* (0.045)**
Constant	3.433* (0.076)**	3.431* (0.076)**	3.976* (0.093)**	0.273* (0.101)**	0.270* (0.101)**	0.518* (0.123)**
Observations	8,530	8,530	8,530	8,530	8,530	8,530
*R*^2^/pseudo *R*^2^	0.0009	0.0009	0.0134	0.0121	0.0122	0.0132

*p < 0.1, **p < 0.05, ***p < 0.01.

### 4.3 Mechanism exploration

Although mediation analyses based on cross-sectional data have inferential limitations, when guided by theory and controlling for relevant confounding variables, they are still widely used for preliminary testing of potential causal pathways among variables (34). To investigate the underlying psychological mechanism by which online learning influences physical activity behavior, this study tested the mediating role of perceived importance of learning. We conducted mediation analysis using the bias-corrected nonparametric bootstrap procedure (1,000 resamples), a method that imposes no stringent distributional assumptions and is among the most robust statistical approaches for testing mediation (33). All models rigorously controlled for sex, age, urban/rural residence, self-rated health, years of education, and survey year to ensure that estimates of the focal pathways represent net effects.

First, path a (online learning → perceived importance of learning) was significant, indicating that participation in online learning effectively increases individuals’ valuation of learning per se. Second, path b (perceived importance of learning → physical activity participation) was also significant, meaning that, controlling for baseline behavior, individuals’ valuation of learning independently predicts their probability of engaging in physical activity.

The indirect effect (a × b) confirmed the statistical significance of the mediation. Meanwhile, the direct effect of online learning on physical activity participation (*p* < 0.01) and the total effect (*p* < 0.01) remained significant, indicating partial mediation. The proportion of the total effect explained by perceived importance of learning was 14.59%. Although this proportion did not reach statistical significance, it indicates that at least 14.6% of the effect is transmitted via an internalized psychological mechanism; in other words, online learning promotes physical activity by enhancing individuals’ generalized valuation of “learning.”

The key mediating role of perceived importance of learning reveals, at the micropsychological level, that in the digital era online knowledge-acquisition behaviors can reshape individuals’ internal value systems and thereby drive offline health practices. This finding elevates the significance of online learning from a mere informational tool to a force for value formation and behavioral cultivation, providing new empirical evidence on how digital interventions can profoundly affect individuals’ lifestyles (see Table 3).

**Table 3:** Bootstrap mediation effect analysis.

Effect path	Coefficient	Bootstrap std. err.	95% CI (percentile)
Path a: X → M			
(Online learning → learning importance)	0.295***	0.020	[0.257, 0.334]
Path b: M → Y			
(Learning importance → sports participation)	0.076***	0.024	[0.026, 0.122]
Direct effect (c′)	0.131***	0.048	[0.041, 0.228]
Total effect (c)	0.154***	0.047	[0.062, 0.252]
Indirect effect (a × b)	0.022***	0.007	[0.008, 0.037]
Proportion mediated	14.59%	10.16	[4.97, 41.14%]

*p < 0.1, **p < 0.05, ***p < 0.01.

### 4.4 Heterogeneity analysis

#### 4.4.1 Educational dimension

We conducted an in-depth examination of the moderating role of education level in the relationship between online learning and sports participation. The statistics in Table 4 indicate that the promotive effect of online learning on sports participation is significant in both the higher-education group (college diploma and above, *N* = 3,461) and the lower-education group (high school and below, *N* = 5,069), with coefficients of 0.131 (*p* < 0.05). This apparently contradictory result actually reveals the nonlinear nature of the educational moderation mechanism. A plausible explanation is that once individuals achieve a basic threshold of education (for example, a high school level), their information access and digital literacy are generally sufficient to benefit from online learning, and additional years of education yield diminishing returns for the effectiveness of online learning. The significance observed in the higher-education group is therefore likely attributable more to structural advantages in knowledge transformation capacity, self-management, and resource accessibility within that cohort, rather than to a simple linear extension of years of education.

**Table 4:** Heterogeneity analysis.

Dimension/statistic	(1) High-edu roup	(2)Low-edu roup	(3) Male	(4) Female	(5) Urban	(6) Rural
Core coefficient						
Online learning	0.131***	0.120***	0.178***	0.111**	0.159***	0.136***
(Robust std. err.)	(0.050)	(0.046)	(0.046)	(0.051)	(0.053)	(0.044)
Key control variable						
Years of schooling	−0.058***	−0.052***	−0.047***	−0.059***	−0.055***	−0.054***
(Robust std. err.)	(0.009)	(0.007)	(0.007)	(0.008)	(0.009)	(0.007)
Sample statistics						
Observations	3,461	5,069	4,697	3,833	3,233	5,297
*R* ^2^	0.0076	0.0023	0.0158	0.0262	0.0142	0.0132
Subgroup definition	College+	High school or below	Male = 1	Male = 0	Urban = 1	Urban = 0

*p < 0.1, **p < 0.05, ***p < 0.01.

#### 4.4.2 Gender dimension

In the heterogeneity analysis, the effect of online learning on physical activity participation exhibits significant subgroup differences. Gender-stratified regressions indicate that the promoting effect of online learning on the frequency of physical activity is greater for males (coefficient = 0.178) than for females (coefficient = 0.111), which may be attributed to males’ greater propensity to engage in sports that rely on knowledge-based learning (e.g., resistance training, specialized technical skills) and their higher self-efficacy in translating online information into concrete action.

#### 4.4.3 Urban and rural dimensions

Urban–rural heterogeneity is also notable: the effect of online learning on urban residents (coefficient = 0.159) is slightly higher than that on rural residents (coefficient = 0.136). Well-developed sports facilities in urban areas may complement the health knowledge provided by online learning, thereby strengthening behavioral translation, whereas limited on-site exercise conditions in rural areas may attenuate the effective conversion of online knowledge into actual participation.

### 4.5 Robustness check

In terms of robustness (Table 5), multiple tests support the stability of the baseline regression results. First, when the dependent variable is replaced by “weekly exercise duration,” the effect of online learning remains positive; although its statistical significance is attenuated after controlling for a range of covariates, this is consistent with expectations because exercise duration is more likely than frequency to be constrained by objective conditions. Second, when “daily participation in online learning” is used as a higher-intensity independent variable, its coefficient reaches 0.306 (*p* < 0.001), substantially larger than that in the base model, After replacing the independent variable with the higher-intensity category “daily online learning,” the effect size (*β*) increased, suggesting that the intensity of online learning engagement may be positively correlated with participation in physical activity, indicating that higher intensity of online learning use is associated with a stronger promoting effect on physical activity participation. Year-specific regressions show coefficients of 0.147 and 0.145 for online learning in 2020 and 2022, respectively, both significant at least at the *p* < 0.05 level, suggesting temporal persistence of its promoting effect. Finally, after replacing the continuous variable for years of education with categorical education levels, the core findings remain unchanged, and it is observed that holding a bachelor’s degree has the largest negative effect on sports participation, which may be related to this group being in the early stages of career development and therefore facing more pronounced time constraints.

**Table 5:** Robustness checks.

Specification	(1) Exercise duration	(2) Daily use	(3) 2020	(4) 2022	(5) Poisson	(6) Edu. measure
Core coefficient						
Online learning	9.458**	0.306***	0.147***	0.145***	0.041***	0.152***
(Robust std. err.)	(3.964)	(0.049)	(0.049)	(0.048)	(0.010)	(0.034)
Key statistics						
Observations	8,530	3,833	4,094	4,436	8,530	8,530
*R*^2^/pseudo *R*^2^	0.0346	0.0225	0.0124	0.0158	—	0.0171
Model details	DV: weekly minutes	IV: daily learning	Only 2020 data	Only 2022 data	Model: for count data	Edu. var: categorical

*p < 0.1, **p < 0.05, ***p < 0.01.

We conducted variance inflation factor (VIF) tests for all explanatory variables to diagnose potential multicollinearity. The VIF results (see footnote) indicate that all variables have VIF values ranging from 1.00 to 1.19, well below the commonly used empirical thresholds in statistics (typically 5 or 10). These findings suggest that there is no severe multicollinearity among the explanatory variables included in the final model, and that the coefficient estimates reported in the baseline regression analyses are reliable and stable.

## 5 Discussion

As digital technology evolves from a ubiquitous communication tool into a systematic online learning platform, a key question arises: can online learning effectively cultivate health literacy and proactive lifestyles among contemporary citizens? Although this study focuses on the specific behavior of sports participation, it offers implications for understanding health equity in the digital era. Our findings indicate that online learning is not only a significant facilitator of youth sports participation (*β* = 0.145, *p* < 0.01; AME = 0.038, *p* < 0.01), but also likely an important driver of the internalization of healthy behaviors and the formation of sustained habits.

This study found that online learning has a significant promotive effect on youths’ participation in physical activity, and this effect becomes more pronounced after disentangling the confounding influence of formal years of education. This observation raises a key dialectical question: are digital human capital and traditional human capital substitutes, complements, or symbionts? The data indicate that formal education functions as a “masking variable” that obscures the independent value of online learning. This suggests that, within an individual-development framework, the two forms of human capital are likely closer to a symbiotic and complementary relationship. Traditional education supplies foundational cognitive capacities and knowledge frameworks, whereas online learning affords individuals the feasibility and motivation to pursue autonomous, continuous, and personalized exploration in health management (35). Reducing online learning to a mere extension or appendage of conventional education may underestimate its potentially transformative role in shaping citizens’ lifestyles during the post-school stage. Our findings indicate that, for young people who have completed basic schooling, online learning is an independent and effective empowerment pathway for constructing a healthy lifestyle.

Does such empowerment operate universally, or does it exacerbate existing inequalities? Heterogeneity analysis shows that the effect is stronger among males and urban youth. For males, the advantage may stem from the coupling between digital-platform content and socially constructed masculine identities: fitness tutorials, activity-data tracking, and performance-analysis content resonate more directly with societal preferences for physical prowess, a sense of control, and fascination with technical mastery. For urban youth, the effect reflects a multiplier between knowledge acquisition and facility support: motivations and knowledge obtained online can be immediately practiced and reinforced in convenient offline venues, communities, and events. The health-promotion outcome is not determined solely by technology but arises from the interaction of technological features, content narratives, and offline social-material infrastructures.

The study further identifies perceived importance of learning as a key psychological mediator through which online learning influences behavior. This implies that the role of online learning transcends a shallow information-transmission logic and engages deeper mechanisms of cognitive rebooting. Through repeated, successful online learning experiences, individuals’ global evaluation of the learning-cognition process may quietly improve and generalize into a positive, domain-general belief in self-development. When individuals come to firmly believe that “learning can change the self,” applying that belief to health behavior becomes a natural extension. Effective interventions therefore do not necessarily require direct inculcation of health knowledge; cultivating a growth-oriented learning mindset can indirectly and systematically enhance individuals’ willingness and self-efficacy in managing their own health.

Of course, digital empowerment is not without limitations. The constraints of this study point to directions for future inquiry: there is substantial heterogeneity in the content and quality of online learning—entertaining short videos and structured MOOCs may produce markedly different effects—and impacts may vary by initial health beliefs, digital skills, or algorithmic recommendation logics in ways not captured here. Future research should move from asking “whether it works” to analyzing “what content works for whom under which conditions.” In addition, whether online incentives can be translated into durable offline habits remains a question requiring long-term follow-up.

For a long time in health-promotion research, instrumental rationality of technological tools and humanistic value rationality have often been set in opposition; technological solutions have been criticized as cold and metric-driven, neglecting intrinsic motivation and complex contexts (36). When online learning engages deep cognition about the value of learning, it can serve as an effective technological intervention. In this study, this primarily manifested as a significant increase in the frequency of physical exercise, although it did not reach statistical significance for the binary outcome of participation versus non-participation. Importantly, individuals can be activated by online learning through internal cognitive motives rather than merely through external constraints or stimuli.

## 6 Conclusion

This study demonstrates that online learning, as an important form of digital human capital investment, exerts a positive effect on sports participation among Chinese youth aged 18–44. The findings confirm that the effects of online learning are not a simple extension of formal education but represent a distinct pathway for promoting health-related behaviors. The main findings can be summarized as follows:

(1) Online learning significantly increases the frequency of physical exercise and positive participation in sports; after adjusting for formal years of education, this effect becomes more pronounced and robust;
(2) The positive effect exhibits clear heterogeneity, being stronger among males and urban youth, yet remaining significant across groups with different educational backgrounds, which suggests a potential universalizing effect;
(3) Mechanism analysis indicates that approximately 14.59% of this effect is mediated by an increased individual perception of the importance of learning, suggesting that online learning operates not only through information transmission but also by reshaping deep cognitive schemas about the value of self-improvement.

The digital era has advanced individuals’ accumulation of value through digital channels for knowledge, skills, and cognitive capacities. Empirical results show that online learning, as an informal and self-directed form of digital human capital investment, yields observable returns for health-related behaviors. Formal education and online learning are not simply substitutes or linear continuations of one another but interact in complex ways across individual development trajectories. Online learning provides youth with a self-development pathway beyond the spatiotemporal constraints of traditional education, enabling proactive and personalized exploration and construction in domains such as health literacy. This offers a new perspective for understanding the synergy between lifelong learning and lifelong health. The confirmation of the mediating role of perceived “importance of learning” aligns with social cognitive theory and self-determination theory, and implies that the role of digital tools should be elevated from mere information delivery to potential drivers of cognitive and motivational change.

Public health actions should move beyond basic provision of digital resources and actively support the development of engaging and scientifically credible online learning resources related to fitness, sports skills, and health literacy. By leveraging cognitive pathways, health communication and intervention design can cultivate individuals’ cognitive habits for actively learning health knowledge and promote narratives across domains that showcase self-improvement and skill acquisition achieved through online learning, thereby advancing national fitness and improving population health.

Although numerous variables were controlled for, several limitations remain, foremost among them the inability to establish definitive causal relationships. Future research could employ longer-term longitudinal or panel data to strengthen causal inference. While we identified a key mediating variable, other psychological or social mechanisms may also contribute and warrant further investigation.

## Data Availability

Publicly available datasets were analyzed in this study. This data can be found here: the datasets for this study can be found in the (China Family Panel Studies) (https://www.isss.pku.edu.cn/cfps/) access requires registration.
